# Endogenous Annexin-A1 Negatively Regulates Mast Cell-Mediated Allergic Reactions

**DOI:** 10.3389/fphar.2019.01313

**Published:** 2019-11-13

**Authors:** Ajantha Sinniah, Samia Yazid, Stefania Bena, Sonia M. Oliani, Mauro Perretti, Rod J. Flower

**Affiliations:** The William Harvey Research Institute, Barts and the London School of Medicine and Dentistry, Queen Mary University of London, London, United Kingdom

**Keywords:** mast cell, Annexin A1, allergic conjunctivitis model, bone marrow-derived mast cells, transgenic mouse model

## Abstract

Mast cell stabilizers like cromoglycate and nedocromil are mainstream treatments for ocular allergy. Biochemical studies *in vitro* suggest that these drugs prevent mast cell degranulation through the release of Annexin-A1 (Anx-A1) protein. However, the direct effect of Anx-A1 gene deletion on mast cell function *in vitro* and *in vivo* is yet to be fully investigated. Hence, we aim to elucidate the role of Anx-A1 in mast cell function, both *in vivo* and *in vitro*, using a transgenic mouse model where the Anx-A1 gene has been deleted. Bone marrow-derived mast cells (BMDMCs) were cultured from wild-type animals and compared throughout their development to BMDMCs obtained from mice lacking the Anx-A1 gene. The mast cell differentiation, maturity, mediator, and cytokine release were explored using multiple biochemical techniques, such as Western blots, ELISA, and flow cytometry analysis. Electron microscopy was used to identify metachromatic granules content of cells. For *in vivo* studies, Balb/C wild-type and Anx-A1-deficient mice were divided into the following groups: group 1, a control receiving only saline, and group 2, which had been sensitized by prior exposure to short ragweed (SRW) pollen by topical contact with the conjunctival mucosae. Allergic conjunctivitis was evaluated blind after 24 h by trained observers scoring clinical signs. Electron micrographs of BMDMCs from Anx-A1-null mice revealed more vacuoles overall and more fused vacuoles than wild-type cells, suggesting enhanced secretory activity. Congruent with these observations, BMDMCs lacking the Anx-A1 gene released significantly increased amounts of histamine both spontaneously as well as in response to Ig-E-FcεRI cross-linking compared to those from wild-type mice. Interestingly, the spontaneous release of IL-5, IL-6, IL-9, and monocyte chemoattractant protein-1 (MCP-1) were also markedly increased with a greater production observed upon IgE cross-linking. This latter finding is congruent with augmented calcium mobilization in BMDMCs lacking the Anx-A1 gene. *In vivo*, when compared to wild-type animals, Anx-A1-deficient mice exposed to SRW pollen displayed exacerbated signs and symptoms of allergic conjunctivitis. Taken together, these results suggest Anx-A1 is an important non-redundant regulator of mast cell reactivity and particularly in allergen mediated allergic reactions.

## Introduction

Allergic eye diseases are common and cause significant morbidity. Several clinical forms are recognized, which include atopic keratoconjunctivitis, vernal keratoconjunctivitis, contact allergy, giant papillary conjunctivitis, and seasonal and perennial allergic conjunctivitis (Saban et al., 2013). Interestingly, the most common type of ocular allergy is seasonal allergic conjunctivitis, which arises upon exposure or sensitization to airborne allergens such as pollen, grass, or ragweed. Despite the different etiologies, the allergic response in the conjunctiva is elicited by contact to an environmental allergen that leads to membrane-bound IgE cross-linking, hence triggering conjunctival mast cell degranulation and release of inflammatory cytokine mediators.

Mast cells are key players in many allergic diseases including dermatitis, asthma, and ocular allergy. They mediate the early-allergic phase reaction through the release of mediators such as histamine, tryptases, prostaglandins, cytokines, and leukotrienes. These inflammatory mediators cause the acute inflammatory symptoms such as conjunctival edema, redness, tearing, and itching. During the late phase, following several hours of allergen exposure, eosinophils are recruited to the conjunctiva.

Annexin-A1 (Anx-A1) is a 37-kDa monomeric member of the annexin superfamily of proteins (13 members in mammals). The protein is present in many differentiated cell types, predominantly abundant in cells of the myeloid lineage including macrophages, mast cells, eosinophils, and neutrophils. Many studies have shown that the synthesis and secretion of Anx-A1 is triggered by glucocorticoids (Goulding et al., 1990; Peers et al., 1993; Ahluwalia et al., 1994; Perretti and Flower, 1996), and this evidence is supported by the strong correlation between peripheral blood Anx-A1 concentrations and plasma cortisol/corticosterone in rodents and man (Perretti and Flower, 1996; Mulla et al., 2005). Glucocorticoids not only stimulate the transcription of Anx-A1 but also induce the release of preexisting pools of Anx-A1 in the cytoplasm *via* a receptor-dependent, non-genomic pathway (Oliani et al., 2000), which is preceded by phosphorylation at key sites in the N-terminus and other sites, catalyzed by protein kinase C (PKC) (Croxtall et al., 2000; John et al., 2003; Solito et al., 2003). Once externalized, Anx-A1 binds to its cognate formyl peptide receptors (FPRs), specifically FPR-L1 (also now known as FPR2 or ALXR in man) in an autocrine or paracrine manner to inhibit cell activation (Gavins et al., 2003; Pieretti et al., 2004; Bena et al., 2012).

Studies by our group and other laboratories, using Anx-A1-null mice, hu-r-Anx-A1, neutralizing antibodies, and antisense agents, have demonstrated that this protein is responsible for many of the acute anti-inflammatory effects of glucocorticoids (D’Acquisto et al., 2008) and that its absence or degradation is implicated in the pathogenesis of asthma and airway hyperactivity (Chung et al., 2004; Ng et al., 2011). Congruently, both full-length Anx-A1 protein and its N-terminal peptide exert potent anti-inflammatory actions in various acute and chronic non-allergic and allergic inflammatory animal models (Bandeira-Melo et al., 2005; D’Acquisto et al., 2008; Lee et al., 2012).

Recently, biochemical and functional studies in human and mouse mast cells using anti-Anx-A1 neutralizing antibodies have indicated that cromones and other “mast cell stabilizers” that are used to treat seasonal ocular allergy exert their inhibitory action on histamine through the release from these cells of the ant-inflammatory protein Anx-A1 apparently by inhibiting a phosphatase (Yazid et al., 2013; Sinniah et al., 2016), thus potentiating the effect of PKC and increasing the amount of phosphorylated protein available for export. We have also reported the existence of a cleaved and inactive form of Anx-A1 in the tears of patients with a severe ocular allergy, known as vernal keratoconjunctivitis (Yazid et al., 2012) and also that Anx-A1 restrains the development of Th17-dependent uveitis in mice (Yazid et al., 2015).

In this study, we use an Anx-A1 null mouse model to explore the role of Anx-A1 in mast cell function *in vitro* as well as in a model of murine allergic conjunctivitis. We provide strong corroborative evidence that Anx-A1 protein is of critical importance to maintain mast cell homeostasis and, hence, to limit allergic inflammation *in vivo* and *in vitro*. We also show that Anx-A1-null mice exhibit vastly increased sensitivity to short ragweed (SRW) pollen compared with wild-type mice.

## Materials and Methodology

### Animals

Female Anx-A1 knockout (Anx-A1^-/-^) BALB/c mice (Hannon et al., 2003) and matching wild-type (WT) mice (6–8 weeks old) were bred in pathogen-free conditions at Charles River, Kent, UK. Animals were kept under standard conditions and maintained according to UK Home Office Regulations (Animal Act 1986), European Union directives, and the Association for Research in Vision and Ophthalmology statement on the experimental use of the animals.

### Generation of Murine Bmdmcs for *in Vitro* Studies

For bone marrow-derived mast cell (BMDMC) generation, femur bones from WT or Anx-A1 knockout (KO) BALB/c mice (three to five mice, which are 4–6 weeks old, Charles River, Kent, UK) were isolated. The progenitor cells were flushed out, collected, and pooled using a sterile protocol and cultured in RPMI 1640 medium (Invitrogen, Paisley, UK) supplemented with 10% FBS, 100 U/ml of penicillin, 100 µg/ml of streptomycin, 4 mM glutamine, 50 µM 2-mercaptoethanol, 0.1 mM non-essential amino acids, 5 ng/ml of r-murine IL-3, and 10 ng/ml stem cell factor (SCF) (PeproTech, London, UK). Cells were assessed and characterized weekly, during the first 4 weeks of culture, for the expression of c-Kit and FcεRI using flow cytometry.

### DNP-IgE/DNP-BSA Activation of BMDMCs

Aliquots of BMDMCs were incubated overnight with anti-mouse monoclonal dinitrophenyl (DNP)-IgE (100 ng/ml; Sigma) to sensitize the cells, and the following day, the cells were activated by adding DNP-BSA (1 µg/ml; Sigma-Aldrich, Dorset, UK). Cell-free supernatants were collected at 1 h to measure histamine and/or PGD2 release. Aliquots were stored at -70°C for subsequent analysis. When drugs or antibodies were tested, these were added to cells 5 min prior to IgE cross-linking.

### Fixation, Processing, and Embedding for Electron Microscopy

Phosphate-buffered saline (PBS) solution containing 0.5% glutaraldehyde and 4% paraformaldehyde was used to fix BMDMCs for 24 h at 4°C prior to the embedment in LR Gold resin (London Resin Co., Reading, Berkshire, UK). The sections were precisely cut using diamond knives on an ultramicrotome (Reichert Ultracut; Leica, Austria) and positioned on nickel grids (EMS) for the immunogold labeling. TEM was used to examine the cellular ultrastructure of mast cells (MCs).

### Calcium Mobilization Assay

BMDMCs were seeded onto 96-well plate and sensitized overnight by incubating at 37°C with 0.5 µg/ml IgE antibody (Serotec, Oxford, UK) in cultured medium (as previously mentioned). The following day, the cells were washed and then incubated with 2 µM Fura 2-AM (Molecular Probes, Paisley, UK) and 1 µM pluronic acid F-127 (Molecular Probes) in an extracellular solution (13 mM glucose, 10 mM HEPES, 147 mM NaCl, 2 mM KCl, 1 mM MgCl_2_, and 2 mM CaCl_2_ at pH 7.3) at 37°C for 1 h in the dark. Consequently, cells were washed thrice with the extracellular solution, before being added to the 96-well plates. The cells were then stimulated with 10 µg/ml anti-IgE. Ionomycin (1 µM) was used as a positive control. The intracellular calcium mobilization was measured by quantifying the ratio of fluorescence emission at 510 nm, prior to sequential excitation at 340 and 380 nm using the NOVOstar microplate reader (BMG LABTECH Ltd., Aylesbury, UK).

### Analysis of Mast Cell Morphology

**Figure 1:**
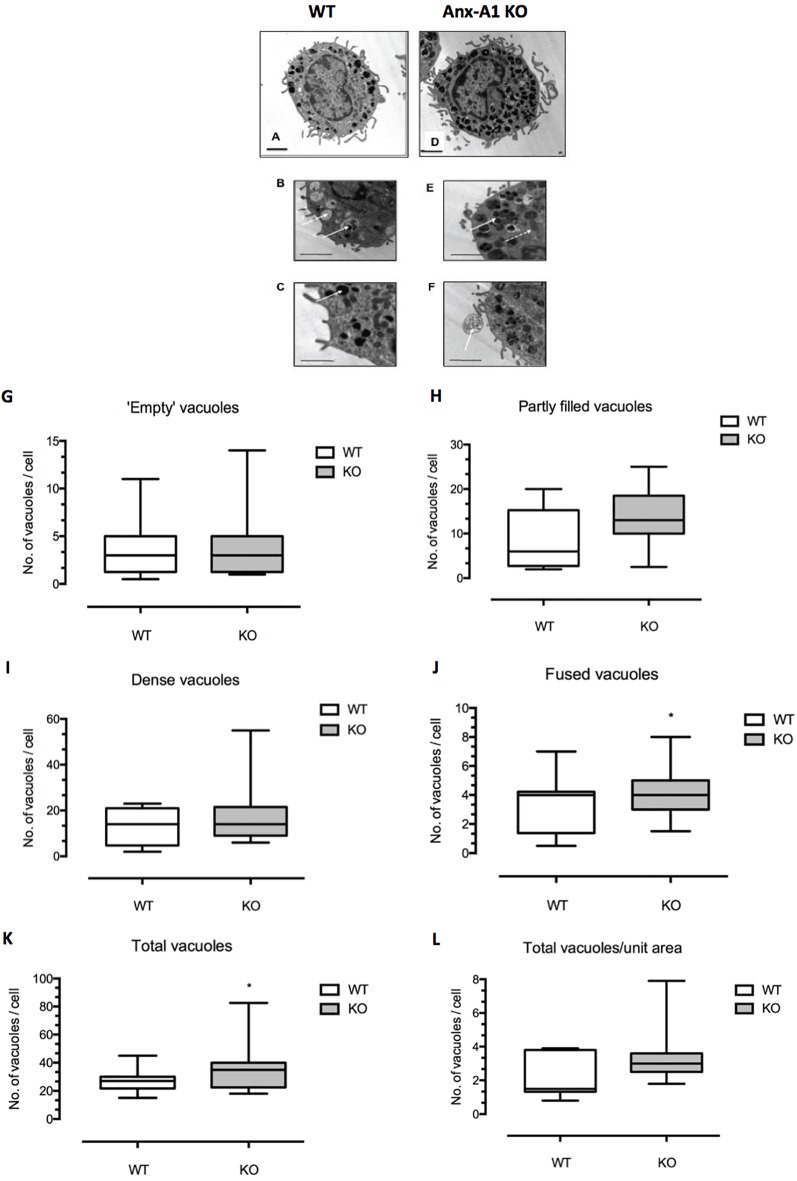
Differences in ultrastructural morphology between wild-type and Anx-A1 null BMDMCs. **(A**–**F)** Electron micrographs of BMDMCs from the wild-type control strain **(A**–**C)** and from the Anx-A1 null line **(D**–**F)**. The morphological features of the vacuoles from the Anx-A1 null BMDMs were shown in **(E** and **F)**. The *white arrow* shown in **(E** indicates fused vacuoles and the *dotted arrow*, mitochondria and extracellular granule remnants are seen in **(F)** (*white arrow*). Whereas “empty” **(B)** (*white arrows*), “partly filled,” and dense vacuoles in **(C)** (*white arrow*) are observed in the BMDMCs from the WT strain. **(G**–**L)** Results of a planimetric analysis of the median number of different vacuoles cells from the two genotypes. There was an overall trend towards a greater number of dense and partly filled vacuoles in the Anx-A1 null strain, although this was not statistically significant. We did, however, observe a significant (*p* 0.05) increase in the number of “fused vacuoles” **(J)** as well as the total number of all vacuoles **(K)** in the Anx-A1 null strain. *Scale bars*, 10 µm * *p* < 0.05 vs WT. Data are expressed as the median ± semi-quartile ranges and were analyzed using the Mann–Whitney test.

Following culture, samples of bone marrow-derived mast cells from both Anx-A1^+/+^ or Anx-A1^-/-^ BALB/c mice were processed as described for analysis by electron microscopy. Twenty-three electron microscopy images of cells were made from the culture of the WT cells and 22 from the Anx-A1 null animals. These were divided into groups and coded. The images were then examined “blind” and the morphology of the cells examined. Only intact mast cells with a clear morphology were used. Those that were located at the edge of the frame were not used since some information could be missing. Altogether 11 cells in the WT group and 12 in the Anx-A1 null group were examined and the number of dense granules, “empty,” “partly filled,” and fused vacuoles were recorded. Since the cells were not perfectly circular, simple measurements of their circumference could not be used to calculate their surface area, so to obtain the figure for total vacuoles/surface area (see **Figure 1**), the surface area of cells were estimated by carefully cutting them out from the printed image and weighing it on an accurate balance. By comparing this to the weight of a known area of paper (calculated in microns), an estimate of the surface area could be obtained. When the data were collated, the study was unblinded and the results sorted into the two groups. Initial statistical tests revealed that the data were not normally distributed, and so the results were analyzed using the Mann–Whitney test.

### Measurement of Histamine Release

Detection and quantification of histamine in the cell supernatant was performed using a commercially available ELISA kit (SPI Bio, Strasbourg, France). The immunoassay was conducted in accordance with the manufacturer’s protocols. The standard curve with a range from 0.39 to 50 nM histamine was prepared using the reagents provided. Optical density was then read at 405 nm within 60 min using a microplate reader (Titertek™, Vienna, Austria).

### Measurement of Cytokines

Milliplex bead arrays (Millipore, UK) were used to determine the cytokine secretion in accordance with the manufacturer’s manual. Culture supernatants (25 µl) were added in triplicate to detect instantaneously the secretion of IL-4, IL-5, IL-6, IL-9, IL-10, TNF-α, and MCP-1.

### Cellular Lysate Collection for Western Blot Analysis

For the determination of STAT-5 protein phosphorylation, BMDMCs were transferred into 1.5-ml Eppendorf tubes, prior to gentle centrifugation at 2,000 rpm for 5 min. The supernatant was then removed and reserved. The pellet was resuspended in 500 µl of lysis buffer containing 1 mM EDTA (for the removal of Anx-A1 attached to the cell membranes), Tris-HCl (pH 8.0), 20 mM Tris-HCl (pH 8.0), 200 mM NaCl, 1 mM protease, and 1 mM phosphatase inhibitors (containing equimolar mixtures of Na_3_VO_4_, β-glycerophosphate, and NaF).

### Determination of STAT-5 Phosphorylation

The total cellular protein was quantified using Bradford protein assay and the extracts were analyzed using conventional semi-dry @estern blotting techniques. Immunodetection was carried out using antibodies recognizing both total STAT-5 protein (polyclonal anti-STAT5 antibody; 1:1,000, Abcam, Cambridge, UK) or STAT-5 phosphorylated on *Tyr* *^694^* (monoclonal rabbit antibody; 1:1,000, Abcam, Cambridge, UK) and monoclonal anti-α-tubulin (1:5,000; Sigma-Aldrich, Poole, UK). As secondary antibody, a horseradish peroxidase-conjugated antibody (1:2,000; Sigma-Aldrich, Poole, UK) was used to detect the relevant bands using the enhanced chemiluminescence technique.

### Experimental Model of Allergic Conjunctivitis

Experimental allergic conjunctivitis was induced in Anx-A1 null (Anx-A1^-/-^) BALB/c mice and WT mice (*n* = 4/experimental group) by topical administration of 1.5 mg SRW pollen antigen suspended in 10 µl of PBS given each day for 8 days prior to the final assessment (Córdova et al., 2014). The conjunctivitis was assessed microscopically, using a clinical scoring scale based upon published grading systems, on the 10th day 1 h after the final application of the antigen. Four clinical signs (conjunctival hyperemia, lid edema, chemosis, and tearing) were monitored and rated by an independent observer in a blinded manner. Each parameter was scored on a scale ranging from 0 to 3, with 0 = absence, 1 = mild, 2 = moderate, and 3 = severe symptoms. The data were expressed as mean ± standard error mean (SEM) for each group. Blood samples were collected through cardiac puncture using a syringe with 10% EDTA. The mice were then sacrificed and the conjunctival tissue and lymph nodes were collected, removed, frozen, and subsequently fixed and sectioned for immunohistochemical assessment.

### Staining of Conjunctival Tissue Mast Cells

Immunostaining of tryptase and Anx-A1 in paraffin sections of conjunctival tissue was performed as follows: The tissue was incubated with 10% normal rabbit serum for 30 min at room temperature, prior to the overnight incubation with rabbit polyclonal anti-tryptase antibodies (Cell Signaling Technology, UK) and Anx-A1 (Zymed, Life Technology, UK) at 4°C. Control tissues were treated with the equivocal concentrations of normal rabbit IgG. Immunoreactivity was detected with biotinylated goat anti-rabbit secondary antibody followed by DAB substrate and counterstained.

### Reagents

Human recombinant Anx-A1 protein was generously supplied by Prof. C. Reutelingsperger from the Cardiovascular Research Institute, Maastricht, Netherlands. SRW pollen (*Ambrosia artemisiifolia*) were purchased from Greer Lab (Lenoir, NC). All the reagents were purchased from Sigma. Antibodies (Stat-5) were purchased from Abcam.

### Statistical Analysis

Data were analyzed using paired or unpaired *t* tests, the non-parametric Mann–Whitney test, or a one-way ANOVA using Prism Software (GraphPad, CA, USA). In all cases, a value of *p* < 0.05 was used as an indicator of a biologically significant effect.

## Results

### Comparison of Morphology and Release of Mediators From BMDMCs Prepared From Anx-A1 Null and Wild-Type Mice

BMDMCs characteristically present a less mature appearance than tissue-derived mast cells (Wernersson, 2014) and generally contain smaller, less dense, partially filled vacuoles. With this knowledge, we examined appearance of cells from both the Anx-A1 null and wild-type strains by electron microscopy and analyzed blindly the number, size, and morphology of granules and vacuoles in cells from the two genotypes.

**Figures 1A–E** show representative electron micrographs of BMDMCs from the wild-type control strain (panels **A**–**C**) and from the Anx-A1 null line (panels **D**–**F**). The former cells generally appeared to be more mature, more numerous and with denser granules. In addition to cellular organelles such as mitochondria (panel **C**, dotted arrow), we observed some fused vacuoles (panel **C**), structures that probably represent extracellular granule remnants (panel **D**), “empty” vacuoles (panel **E**), and partly filled and “dense” granules (panel **F**) in cells from both genotypes. The presence of fused vacuoles and granule remnants may be indicative of secretory activity (Azouz et al., 2014).

Panels **G**–**L** show the results of a more formal analysis of the median number of vacuoles cells from the two genotypes. There was an overall trend for a greater number of dense granules and partly filled vacuoles in Anx-A1 null cells, although this was not statistically significant. A significant increase in the number of “fused vacuoles” (panel **J**) as well as the total number of vacuoles (panel **K**) was quantified in Anx-A1 null BMDMCs.

### Anx-A1 Regulates Mast Cell Maturation and Spontaneous Degranulation

**Figure 2:**
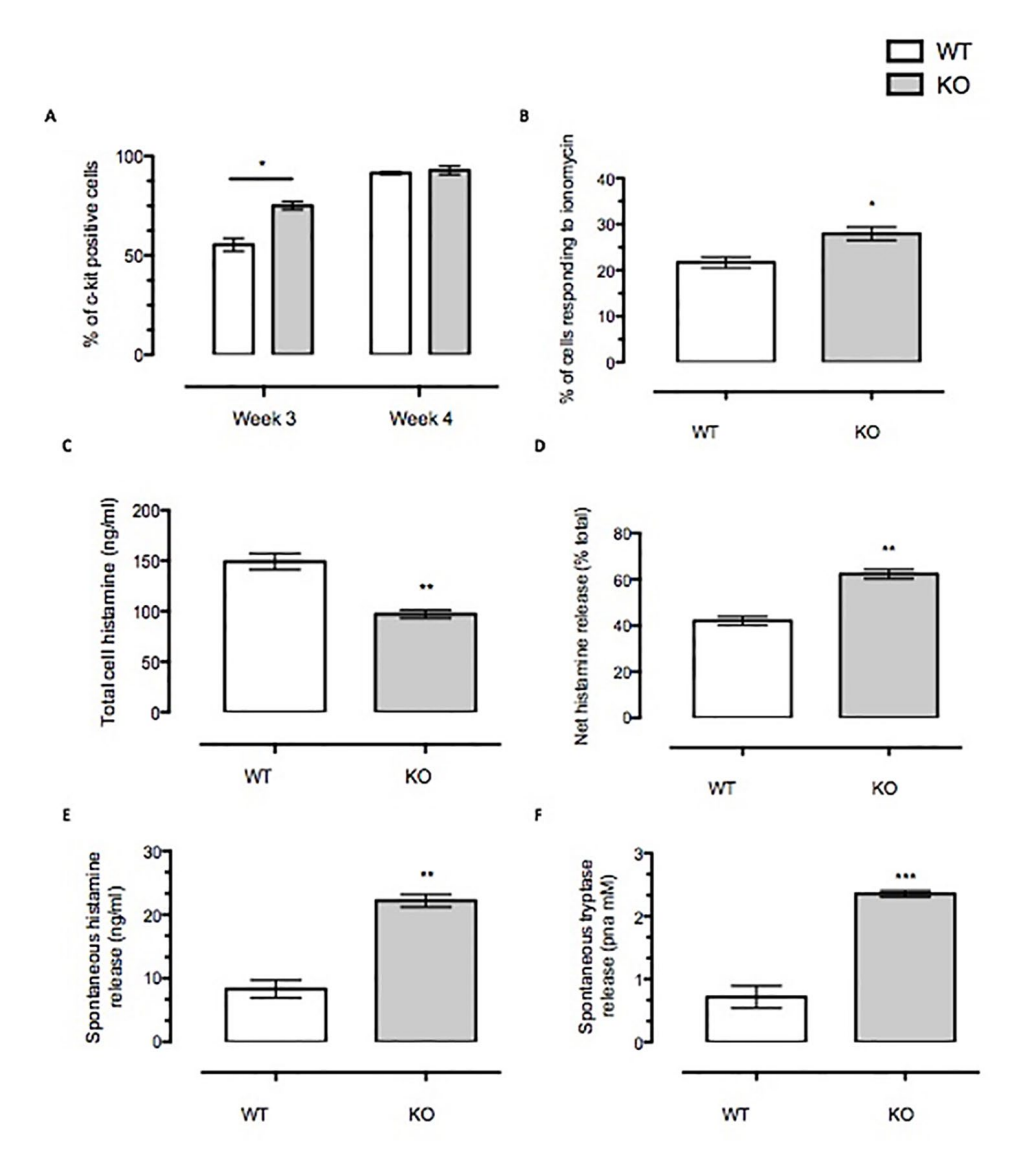
Anx-A1 regulates mast cell maturity and spontaneous degranulation. BMDMCs were prepared from WT and Anx-A1 KO mice as described, sensitized with anti-DNP-IgE (100 ng/ml; Sigma), and challenged with DNP-BSA (1 µg/ml; Sigma Aldrich, Dorset, UK). **(A)** At week 3 culture, the Anx-A1 KO BMDMCs expressed approximately 30% more c-Kit-positive cells as compared to their WT counterparts. At week 4 culture, both WT and Anx-A1 KO BMDMCs reached 90% c-Kit-positive cells. **(B)** BMDMCs from the Anx-A1 KO mice are significantly (*p* 0.05) more sensitive to ionomycin (1 µM) when compared to WT. **(C)** The total intracellular histamine level in Anx-A1^-/-^ BMDMCs is significantly (*p* 0.05) lower than the WT cells; however, **(D)** demonstrates that these cells undergo higher net histamine release in comparison to the WT control (*p* 0.05). **(E** and **F)** Unstimulated Anx-A1 KO BMDMCs spontaneously release more histamine and tryptase when compared to WT BMDMCs. * *p* < 0.05; ** *p* < 0.01 and *** *p* < 0.001 vs WT. Data are expressed as mean ± SEM and analyzed using the two-tailed *t* test.

We also assessed other markers of maturation in BMDMCs such as c-Kit staining and Ca^2+^ fluxes provoked by ionomycin challenge (**Figure 2**). Our data presented suggest that Anx-A1 null cells mature more rapidly than their WT controls in that a greater percentage of these cells express c-Kit staining and also respond to ionomycin stimulation at 3 weeks (**Figures 2A, B**). Taken together, these data suggest that presence of Anx-A1 regulates the rate at which these cells mature.

We have also determined the spontaneous release of histamine and tryptase from BMDMCs. **Figure 2C** shows that Anx-A1 null BMDMCs contained significantly less (∼35%, *p* < 0.05) total cell histamine than the WT controls. However, their net histamine release was significantly higher (∼40%, *p* < 0.05) than the WT BMDMCs (**Figure 2**) upon stimulation with IgE antigen. Cells prepared from Anx-A1 KO mice displayed augmented spontaneous release of histamine (∼30%) and tryptase (+300%) than their WT counterparts (**Figures 2E, F**).

### Spontaneous and Ige-Challenged Anx-A1^-/-^ Bmdmcs Release Pro-Inflammatory Cytokines

**Figure 3:**
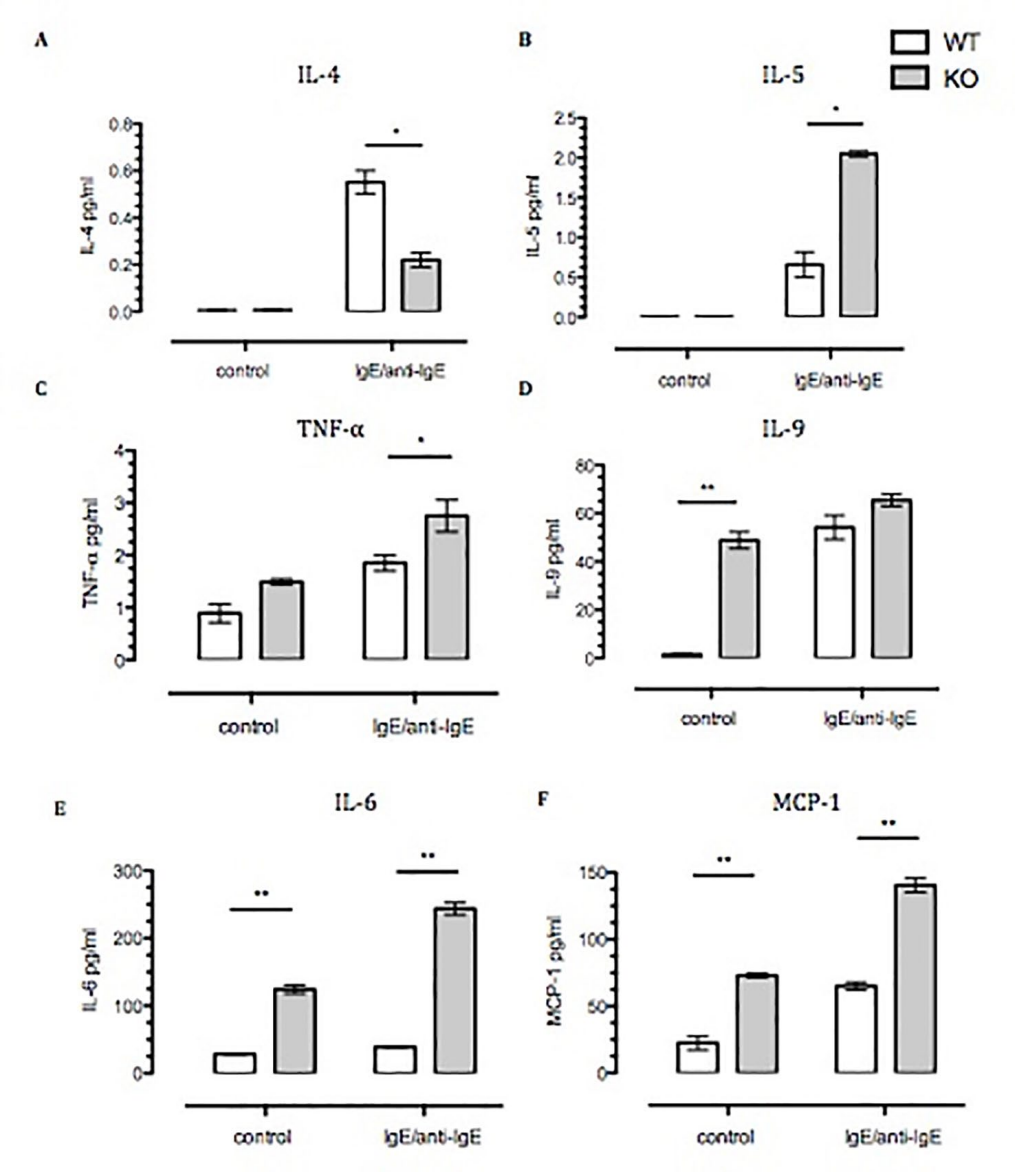
Spontaneous, and Ig-E challenged, release of pro-inflammatory cytokines by Anx-A1^-/-^ BMDMCs. BMDMCs were prepared from WT and Anx-A1 KO mice as described, sensitized with anti-DNP-IgE (100 ng/ml; Sigma), and challenged with DNP-BSA (1 µg/ml; Sigma Aldrich, Dorset, UK). **(A** and **B)** Unstimulated WT and Anx-A1 KO BMDMCs did not express IL-4 and IL-5. But upon sensitization, the expression of IL-4 was significantly (*p* 0.05) decreased while IL-5 expression was significantly (*p* 0.05) increased in Anx-A1 KO BMDMCs in comparison to their WT counterpart. **(C)** BMDMCs lacking the Anx-A1 gene release TNF-α in both unstimulated and stimulated conditions. **(D**–**F)** Unstimulated Anx-A1 KO BMDMCs spontaneously release IL-9, IL-6, and MCP-1 when compared to WT cells. The level of IL-9 was increased in both WT and KO BMDMCs upon stimulation, but the expression of IL-6 (*p* 0.001) and MCP-1 (*p* 0.05) was significantly increased in the Anx-A1 KO BMDMCs compared to the WT cells. * *p* < 0.05 and ** *p* < 0.01 vs WT. Data are expressed as the mean ± SEM and analyzed using the one tailed *t* test.

Next we focused on cytokine release, using a strong stimulation as the one attained with IgE–anti-IgE cross-linking. While there was no difference in the spontaneous release of IL-4, IL-5, or TNF-α (**Figures 3A–C**) between BMDMCs from the two genotypes, there were significant differences between their responses to IgE stimulation. The stimulated IL-4 release was significantly reduced in Anx-A1 null cells (by ∼60%); however, the release of the other cytokines was markedly enhanced (**Figures 3D–F**). Of interest, the spontaneous basal release of IL-6, monocyte chemoattractant protein-1 (MCP-1), and IL-9 was increased in the absence of Anx-A1, indicating fundamental modulatory functions also at the basal state.

### Anx-A1 Regulates P-STAT5

**Figure 4:**
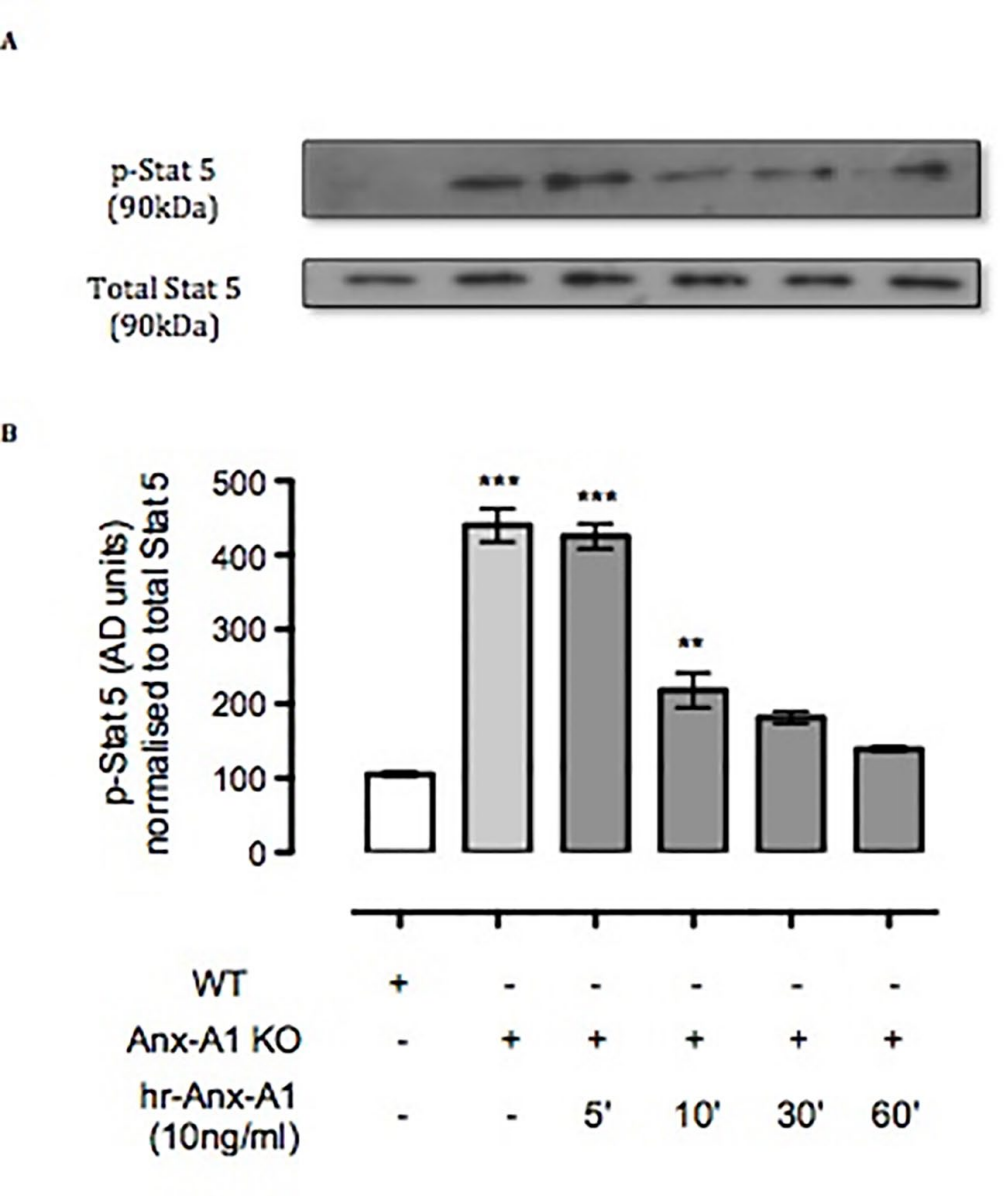
Anx-A1 regulates phospho-Stat 5. BMDMCs were prepared from WT and Anx-A1^-/-^ mice as previously described, sensitized with anti-DNP-IgE (100 ng/ml; Sigma), and challenged with DNP-BSA (1 µg/ml; Sigma Aldrich, Dorset, UK). Analysis of Stat-5 phosphorylation in WT and Anx-A1 KO BMDMCs in the presence of human recombinant Anx-A1 (10 ng/ml) at various time points was accomplished by Western blot analysis **(A)**. Following challenge, KO BMDMCs expressed more than fourfold increase of Stat-5 phosphorylation (*p* 0.001) in comparison with WT cells. However, hr-Anx-A1 (10 ng/ml) reversed this effect in a time-dependent manner. **(B)** Densitometry values of three independent experiments were expressed as the mean percentage of WT control values ± SEM and analyzed by one-way ANOVA. Phospho Stat-5: **p 0.01 and ***p 0.001 vs. WT.

Another master regulator of mast cell differentiation and survival is Stat-5 (Shelburne et al., 2003), which recently has been linked to the development of both spontaneous and allergen-induced dermatitis (Ando et al., 2014). Thus, we ascertained the expression of Stat-5 in BMDMCs derived from WT and Anx-A1^-/-^ mice. Western blot analysis (**Figures 4A and B**) shows a 4.5-fold increase in the expression of Stat-5 in BMDMCs from Anx-A1^-/-^ mice when compared to their WT counterparts. Such a response was rescued by the application of exogenous Anx-A1 (10 ng/ml) to mast cells cultured from Anx-A1-null mice as assessed over a 60-min time course (**Figure 4B**).

### Anx-A1 KO Mice Develop Enhanced Allergic Conjunctivitis to SRW

**Figure 5:**
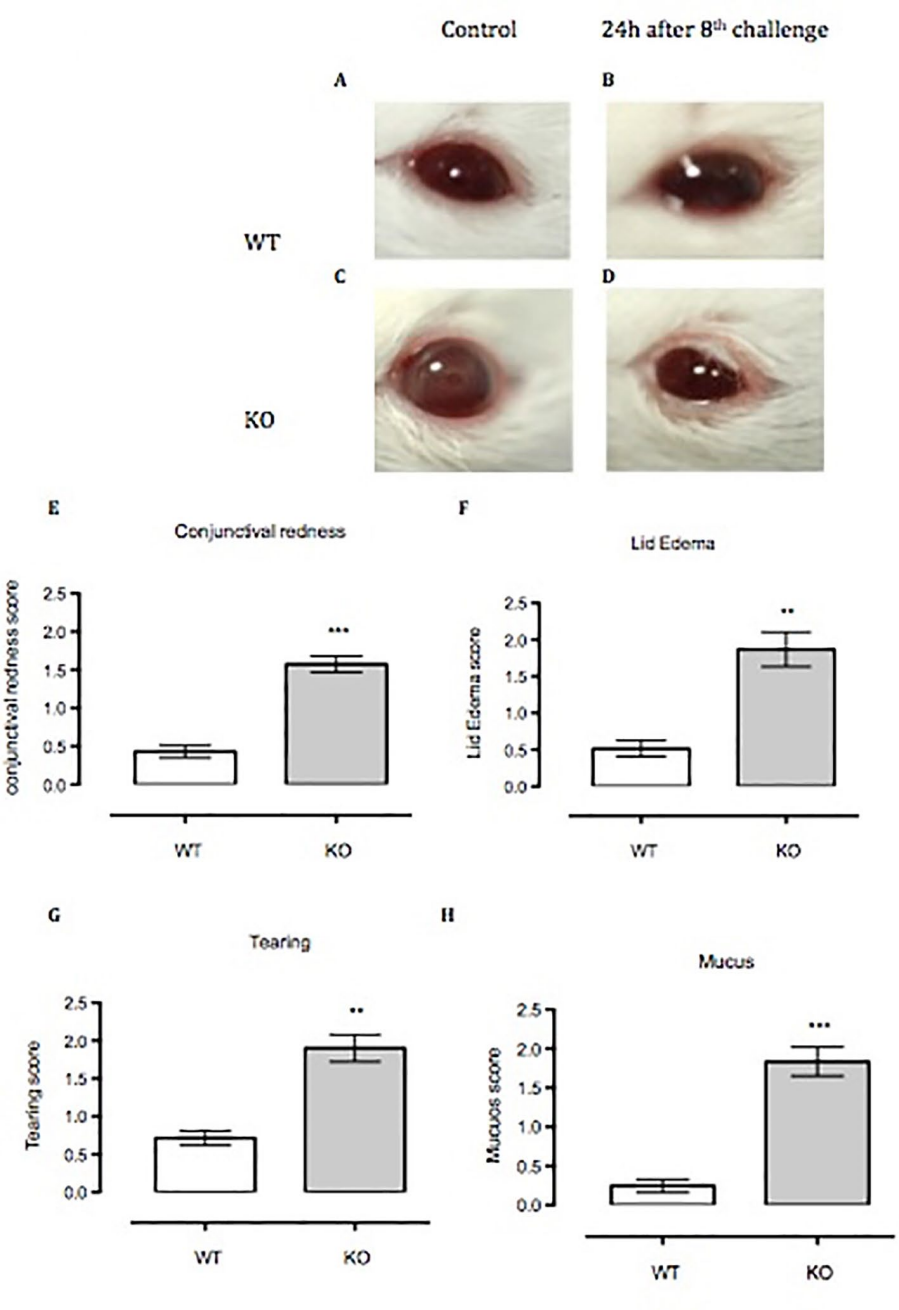
Anx-A1^-/-^ mice develop enhanced allergic conjunctivitis upon SRW pollen exposure. **(A**–**C)** Anx-A1^-/-^ and WT mice were topically challenged for 8 days with SRW pollen or sterile PBS (control group) and the clinical scores were evaluated on the final day 20 min post-challenge. Anx-A1^-/-^ **(D)** mice exposed to SRW pollen exhibited pronounced clinical signs of conjunctivitis when compared to WT **(B)**, including a significant increase of conjunctival redness **(E)**, lid edema **(F)**, tearing **(G)**, and mucus **(H)**. Data were expressed as the mean ± SEM and analyzed by Student’s *t* test. *p 0.05, **p 0.01, ***p 0.001.

Anx-A1 KO mice challenged for 8 days with topical SRW allergen presented moderate to severe clinical signs of conjunctivitis compared to respective controls (**Figures 5A–D**). We measured clinical scoring between WT and Anx-A1 KO mice, 20 min after the eighth topical challenge with the pollen. Anx-A1 KO mice exhibited higher clinical scoring for conjunctival redness (*p* < 0.01), lid edema (*p* < 0.01), tearing (*p* < 0.01), and mucus secretion (*p* < 0.001) when compared to WT mice (**Figures 5E–H**), suggesting that the mast cells from the transgenic animals are more numerous and/or prone to degranulate in response to antigen.

### Mast Cell Numbers Are Increased in Anx-A1 Null Conjunctival Tissue

**Figure 6:**
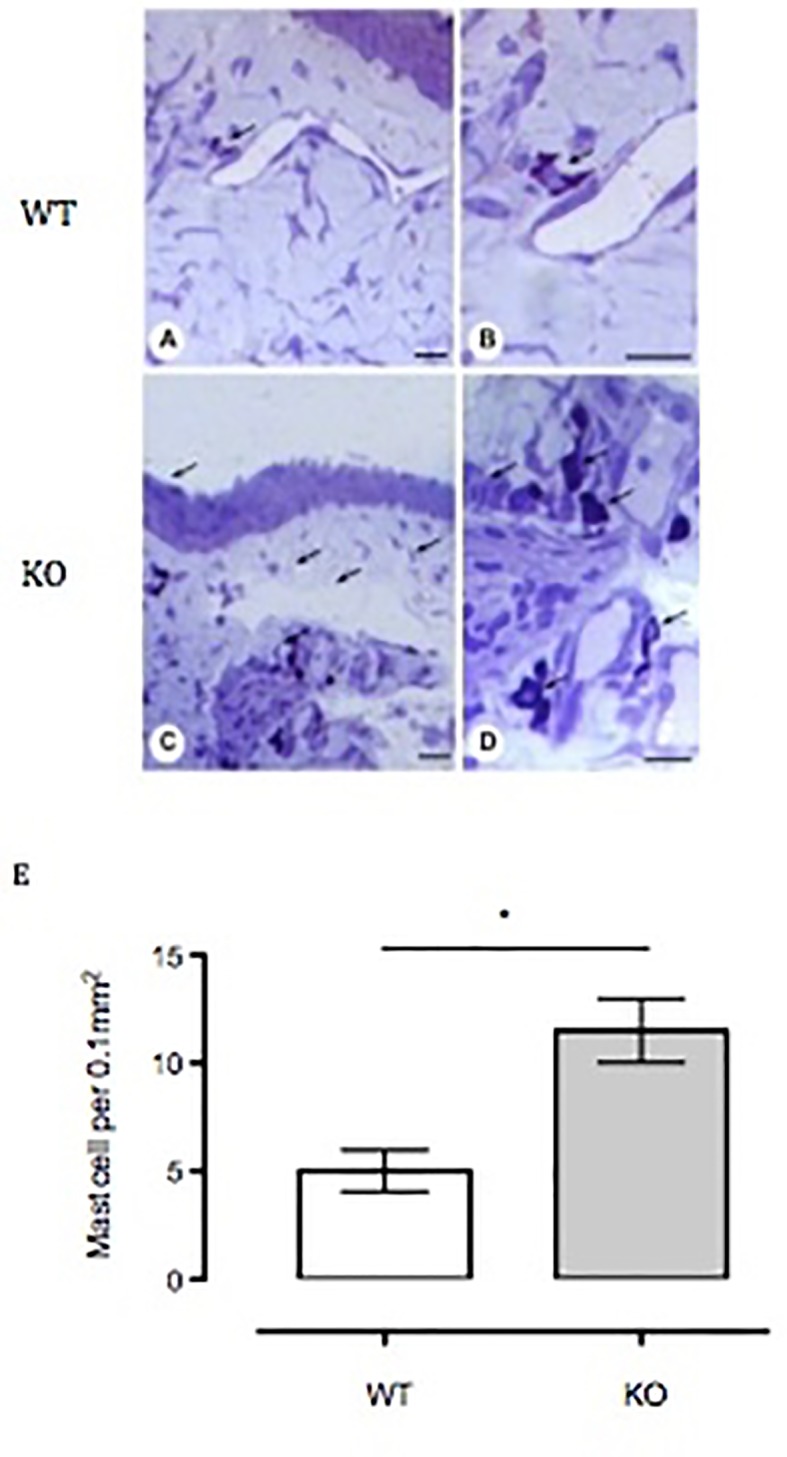
Influx of mast cells into the bulbar conjunctival tissue of Anx-A1-null mice. Histological analysis of conjunctival tissue 24 h post-SRW pollen-challenge indicates an increased influx of mast cells in the Anx-A1 KO mice **(C** and **D)** as compared to WT **(A** and **B)**. There were a higher proportion of degranulated mast cells observed in the conjunctival tissue of Anx-A1^-/-^ mice, whereas intact metachromatic granules were observed in the WT mice. Quantitative analysis of the number of mast cells in both WT and Anx-A1^-/-^ was shown in **(E)**. Data represent the mean ± SEM of the number of cells per 0.1 mm^2^ for each group (each group = 4 mice). Stain: Toluidine blue. *Scale bar*, 10 µm. Data expressed as the mean ± SEM and analyzed by Student’s *t* test. *p 0.05 vs WT.

To assess mast cell numbers, we analyzed the bulbar conjunctival tissue obtained from WT and Anx-A1 KO mice. **Figure 6** shows that bulbar conjunctival tissue from Anx-A1^-/-^ mice (**Figures 6C, D**) presents significantly increased infiltration of mast cells when compared to WT mice (**Figures 6A, B**). Histopathological analyses (**Figure 6E**) revealed a twofold increase in intact conjunctival mast cells in Anx-A1^-/-^ mice when compared to WT mice (*p* < 0.05).

## Discussion and Conclusion

Evidence from recent biochemical and imaging studies conducted in our laboratory have shown that Anx-A1 is crucial for the acute inhibitory actions on mast cells exerted by cromone and other anti-allergic drugs (Yazid et al., 2013; Sinniah et al., 2016). This mechanism is shared with other cell types, as demonstrated in U937 cells (Yazid et al., 2009) and PMN (Yazid et al., 2010). Here we refocus on the mast cell and demonstrate the non-redundant importance of Anx-A1 as an endogenous negative regulator of mast cell function, with implications for the physiopathological properties of this cell type.

Mast cells are pivotal players of the ocular allergic inflammation response. The presence of an expanded mast cell population, and the subsequent enhanced release of preformed and newly synthesized mediators in the conjunctiva, is characteristic of ocular allergic reactions (Leonardi, 2002). Similar studies also show that mast cell numbers were increased in glaucoma patients, strengthening the notion of important players across eye disease board (Chang et al., 2009). Thus, the involvement of mast cells has also been evaluated in uveitis (da Silva et al., 2011) and atopic keratoconjunctivitis (Udell et al., 2002). Interestingly, bulbar conjunctival tissue obtained from the Anx-A1 KO mice indicated enhanced numbers of mast cells when compared to the WT mice. These data resonate with a recent investigation which reported that Anx-A1 KO mice exhibited increased number of mast cells in the lamina propria of conjunctiva compared to sham WT, as detected by histopathological studies (Gimenes et al., 2015).

Since it has been suggested that cytoplasmic Anx-A1 is crucial for granule formation in the mast cells (Kim et al., 2008), we investigated whether there were structural differences between BMDMCs from Anx-A1 null and WT mice. Secretory proteins in these cells are synthesized and sorted into transport vesicles that bud off and fuse into condensed vacuoles, which then become mature secretory vesicles (Wang et al., 2002). Transmission electron microscopy analysis demonstrates that unstimulated BMDMCs lacking the Anx-A1 gene have an increased total number of vacuoles as well as fused vacuoles when compared to the WT control cells. Our data indicate that Anx-A1 KO BMDMCs undergo spontaneous activation, as evidenced by increased secretory activity, suggesting that, indeed, Anx-A1 plays a critical role in modulating biogenesis and turnover of secretory vesicles in mast cells. Even though the release of histamine and tryptase is commonly used as an indicator of mediator release from mast cells, it is unclear whether this accurately reflects the behavior of all secretory granules in mast cells.

To ascertain whether spontaneous degranulation of Anx-A1 KO BMDMCs could be attributed to the phenotypic maturity, we monitored c-Kit receptors in these cells during culture. c-Kit (CD117) is a growth factor receptor which signal to regulate multiple physiological functions of mast cells, including cell growth, differentiation, maturity, and survival as well as mast cell migration or chemotactic homing (Gilfillan and Beaven, 2011). The binding of the c-Kit receptor by its specific ligand, SCF, stimulates the catalytic activity and downstream signaling cascade, which initiates these biological effects. SCF itself can regulate mast cell degranulation, cytokine production (Bischoff and Dahinden, 1992; Hundley et al., 2004), and secretory functions under experimental conditions. Our study revealed that the BMDMCs from Anx-A1 KO mice exhibit a significant increase of c-Kit-positive mast cells at week 3 when compared to those from the WT mice.

c-Kit receptor expression on mast cells denotes a functionally defined subset that is more responsive to degranulation. Indeed, evidence from biochemical and imaging studies described here have further confirmed that release of Anx-A1 is critical to exert an inhibitory tone in mast cells. BMDMCs from the Anx-A1 KO mice therefore appear to differentiate at an earlier time point as compared to BMDMCs from WT mice. Upregulation of c-Kit receptor expression on activated mast cells correlates with increased mediator release.

Another important parameter involved in mast cell exocytosis is Ca^2+^ mobilization. Influx of Ca^2+^ into the cytosol is essential also for other cellular activities, including proliferation, adhesion, gene expression, and migration (DiMaio et al., 2009). Our study revealed that higher percentage of Anx-A1 null BMDMCs respond to ionomycin in comparison to their WT counterpart. Sustained increase of cytosolic Ca^2+^ ions is crucial for mast cell activation (Ma and Beaven, 2011).

Another important regulator of mast cell maturation is the signal transduction protein, Stat-5. Stat-5 activation induced by IL-2 is crucial for Th-2 cytokine differentiation *in vitro* (Paul and Zhu, 2010). Our results show that Anx-A1 null BMDMCs express higher amounts of Stat-5 compared to WT BMDMCs. Interestingly, even though these cells lack the Anx-A1 gene and thus cannot release Anx-A1, yet they retained their sensitivity to hr-Anx-A1, which attenuated the overexpression of Stat-5. These data corroborate our previous finding whereby exogenous Anx-A1 inhibited expression of histamine and PGD_2_ in BMDMCs from Anx-A1 KO mice (Yazid et al., 2013). This inverse relationship between the level of Anx-A1 and the activation of Stat-5 may underpin the anti-allergic property of this protein.

It has been well documented that skewing of Th2 cytokines (IL-4, IL-5, IL-6, IL-9, and IL-13) orchestrates allergic responses (Barnes, 2001; Gilfillan and Tkaczyk, 2006). IL-4 promotes Ig-E production, which binds to the FcεRI receptor on mast cells (Brown et al., 1987; Boyce, 2003), whereas IL-5 and IL-9 induce tissue eosinophilia and mast cell hyperplasia (Romagnani, 2002; Sabatino et al., 2012; Tete et al., 2012), respectively. Ig-E challenge significantly increased the levels of IL-5, IL-6, and MCP-1 in Anx-A1^-/-^ BMDMCs when compared to the WT control, suggesting that lack of endogenous Anx-A1 in the Ig-E-challenged group exacerbated the humoral response by increasing Th2 cytokine levels. Interestingly, unstimulated Anx-A1^-/-^ BMDMCs secrete increased amounts of IL-6, IL-9, and MCP-1, emphasizing the importance of endogenous Anx-A1 in restraining spontaneous activation of mast cells. Other laboratories have shown that pro-inflammatory cytokines such as IL-6 and TNF-α are elevated in lung fibroblast cells lacking the endogenous Anx-A1 gene (Yang et al., 2006; Jia et al., 2013). In our experimental model, we observed that IL-4 levels were increased in the Ig-E-challenged WT BMDMCs compared to the sham control; however, IL-4 expression was significantly reduced in stimulated Anx-A1^-/-^ BMDMCs. This observation is in agreement with that of Gimenes *et al*, who found reduced IL-4 levels in the lymph nodes of ovalbumin (OVA)-challenged Anx-A1^-/-^ mice (Gimenes et al., 2015). Elevated IL-4 expression downregulates c-Kit expression in mast cells (Sillaber et al., 1991; Nilsson et al., 1994; Thienemann et al., 2004). Moreover, BMDMCs cultured with IL-4 show decreased c-Kit and FcεRI expression in comparison with human mast cells (Shelburne et al., 2003; Mirmonsef et al., 1999; Ryan et al., 1998). Since we have demonstrated here that BMDMCs lacking the endogenous Anx-A1 exhibit increased phenotypic maturity as assessed by enhanced expression of the c-Kit receptor, it could be possible that these cells produce lesser amount of IL-4. Mast cells are known to maintain a constitutive presence of functional IL-4 transcripts, but not IL-4 cytokines. However, the constitutive presence of IL-4 transcripts is sufficient for the rapid production of IL-4 cytokines upon mast cell activation. Since the differences in phenotype we observed in BBMCs derived from null mice compared to WT, it may be that they have altered constitutive transcripts for IL-4 or in response to antigen challenge or a limited production of IL-4 cytokine due to the high IgE level at baseline. While we think it is an interesting finding, we have not investigated it further in this study.

Data from our *in vitro* studies suggest a protective role for Anx-A1 in the allergic response through negative regulation of pro-inflammatory cytokine profiles. Therefore, we concluded our study by testing this *in vivo*. WT and Anx-A1^-/-^ mice exposed to SRW pollen displayed pathological hallmarks of conjunctivitis, including intense hyperemia, lid edema, mucous secretion, and tearing of the eye, in comparison to their respective controls. Our data show that the ensuing allergic conjunctivitis was more severe in Anx-A1^-/-^ mice when compared to their WT counterpart. This is in line with the other findings where the allergic response in a model of OVA-induced allergic conjunctivitis was greatly diminished in WT mice treated with the bioactive Anx-A1 peptide, Ac_2-26_, further reinforcing the evidence for a beneficial effect of Anx-A1 in intraocular inflammation (Gimenes et al., 2015). Similar findings were also reported in an OVA-induced asthma model, in which PGD_2_ levels were significantly reduced upon treatment with Anx-A1 peptide (Wang et al., 2011), and furthermore, Anx-A1^-/-^ mice exhibited increased airway hyper-responsiveness and an increased influx of leukocytes in lungs and bronchoalveolar lavage (Ng et al., 2011). A very recent study has demonstrated that Anx-A1 exert anti-allergic response in OVA-induced atopic dermatitis model by regulating the production of cytokines and increasing mast cells activation, hence leading to the exacerbation of experimental atopic dermatitis (Parisi et al., 2019).

Collectively, our data here clearly reveal that Anx-A1 is a key determinant in mast cell biology and indicates a critical regulatory role for endogenous Anx-A1 in the maturation and function of mast cells as well as the development of allergic inflammatory responses. The release of Anx-A1 may be responsible for the observed actions of glucocorticoids on this process.

## Data Availability Statement

All datasets generated for this study are included in the article/supplementary material.

## Ethics Statement

The animal study was reviewed and approved by UK Home Office Regulations (Animal Act 1986), European Union directives and the Association for Research in Vision and Ophthalmology statement on the experimental use of the animals.

## Author Contributions

AS, SY, SB, and SO performed the experimental work. AS and SY wrote the manuscript; AS coordinated the submission. MP provided general scientific overview and critically revised the manuscript. RF provided general scientific oversight and input and revised the final manuscript. All authors have read and approved the final manuscript.

## Funding

This work was funded by The Wellcome Trust (award number 085903). AS was supported by the Government of Malaysia.

## Conflict of Interest

The authors declare that the research was conducted in the absence of any commercial or financial relationships that could be construed as a potential conflict of interest.
